# Epigenetic insights into prostate cancer: exploring histone modifications and their therapeutic implications

**DOI:** 10.3389/fonc.2025.1570193

**Published:** 2025-04-28

**Authors:** Bunty Sharma, Himanshu Shekhar, Anidrisha Sahu, Damandeep Kaur, Shafiul Haque, Hardeep Singh Tuli, Himanshu Sharma, Ujjawal Sharma

**Affiliations:** ^1^ Department of Biotechnology, Graphic Era (Deemed to be University), Dehradun, Uttarakhand, India; ^2^ Department of Human Genetics and Molecular Medicine, Central University of Punjab, Bhatinda, India; ^3^ University Center for Research and Development (UCRD), Chandigarh University, Mohali, Punjab, India; ^4^ College of Nursing and Health Sciences, Jazan University, Jazan, Saudi Arabia; ^5^ School of Medicine, Universidad Espiritu Santo, Samborondon, Ecuador; ^6^ Department of Bio-Sciences and Technology, Maharishi Markandeshwar Engineering College, Maharishi Markandeshwar (Deemed to be University), Mullana, India; ^7^ Department of Biochemistry, All India Institute of Medical Sciences, Bathinda, India

**Keywords:** prostate cancer, epigenetics, histone modification, therapeutics, epigenetic modifications

## Abstract

Prostate cancer is one of the most prevalent malignancies globally. This cancerous condition originates within the prostate gland, an integral part of the male reproductive system. The molecular mechanism underlying cancer is among the key areas of research in the scientific community. Cancer, being a multifactorial disease, is controlled by many factors ranging from environmental to genetic to epigenetic factors. Epigenetic regulation holds a crucial role in tumorigenesis and its progression. Epigenetics refers to alterations in the genome that happen without any changes to the DNA sequence itself; they may be triggered by multiple factors ranging from environmental to dietary factors. It includes methylation of DNA and histone modifications. Histone modifications, including histone methylation, histone acetylation, and histone ubiquitination, play a crucial role in the pathogenesis and progression of prostate cancer. These epigenetic modifications via transcriptional regulation affect key cellular processes and are thus implicated in prostate cancer and other cancers. These epigenetic markers could be used as both diagnostic and prognostic markers and also could be used as novel therapeutic targets against prostate cancer and other malignancies. Here in this review article, we have summarized different histone modifications and their mechanistic and therapeutic implications in prostate cancer.

## Graphical Abstract

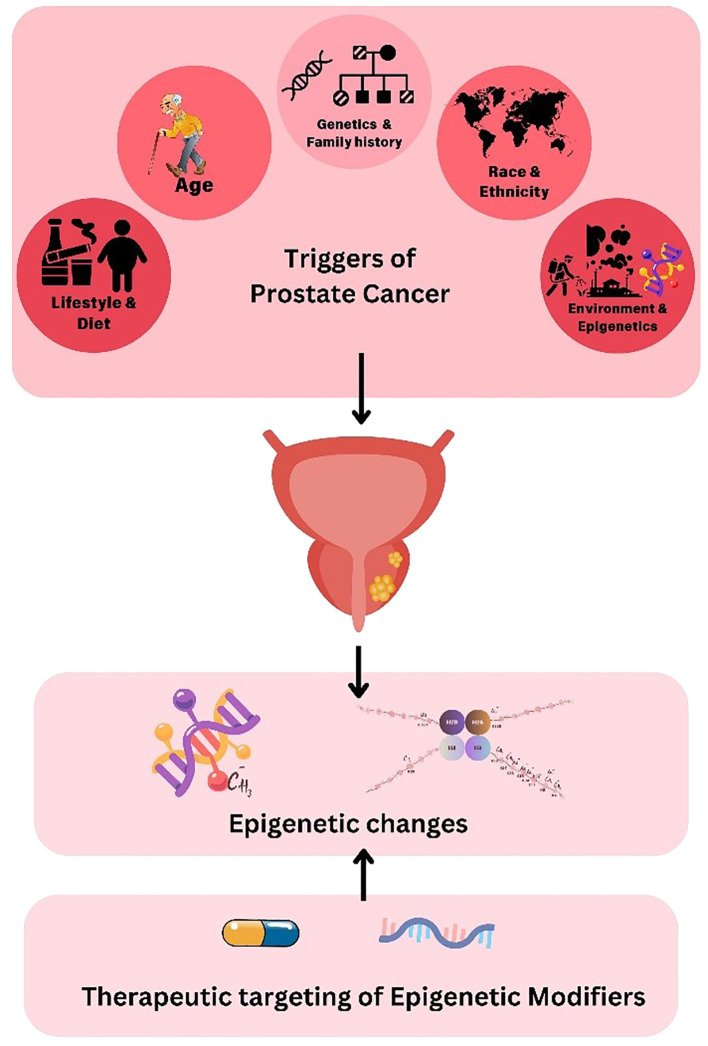

## 1 Introduction

Prostate tumor stands as the second most prevalent diagnosed malignancy and the fifth most common cause of mortality due to cancer globally, with an approximation of new cases reaching 1.4 million and a death toll reaching 3.75 lakhs in 2020 alone (1). The incidence of prostate cancer is rising among older men. Prostate cancer is associated with many risk factors including age, race, ethnicity, family history, and other factors such as occupational, environmental, and dietary factors (2, 3). In the early stages, prostate cancer may be undetected due to its asymptomatic nature. Delayed diagnosis is a major reason for the high mortality of prostate cancer. Presently, prostate cancer diagnosis mostly relies on prostate-specific antigen (PSA) screening and biopsy techniques, which are not very efficient and may lead to under- or over-diagnosis of the disease. Also, current treatment strategies such as chemotherapy and hormone therapy are associated with risk and side effects. Therefore, researchers are looking for a more precise novel diagnostic and prognostic biomarker and novel therapeutic target for prostate cancer. Epigenetic markers are among such novel markers that are extensively explored nowadays and are reported to be implicated in multiple cancers. Epigenetics plays a key role in tumorigenesis and the progression of tumors, and epigenetic markers are emerging as a novel hallmark of cancer (4). Epigenetic modifications such as methylation of DNA as well as modifications in histone bring out transcriptional activation or suppression and thus modulate key cellular processes. Methylation of DNA is directly involved in gene expression regulation and could be used as both diagnostic and prognostic biomarkers of prostate cancer (5). However, histone modifications are also an important epigenetic marker, which are found to be associated with many cancers including prostate cancer (6). Among major histone modifications are histone methylation and acetylation. Histone acetylation entails attaching an acetyl group to lysine residues located on histone tails (7). This process is facilitated by two key enzyme groups: histone acetyltransferases (HATs) and histone deacetylases (HDACs). A distinct acetylation pattern brings transcriptional changes, thus affecting key pathophysiological pathways of prostate cancer. However, histone methylation refers to methyl group addition at the basic amino acid residue of the histone chain, and it is mediated by two enzymes: histone methyltransferases (HMTs) and histone demethylases (HDMs). Differential methylation patterns as well as differential expression patterns of HMTs and HDMs are reported in prostate cancer, and these could be used as novel biomarkers as well as therapeutic targets (8). In addition to acetylation and methylation, other histone modifications like phosphorylation and ubiquitination have also been reported to be involved in prostate cancer (9, 10). In this article, we have thoroughly reviewed different histone modifications involved in prostate cancer, giving a holistic view of current scientific knowledge of the molecular mechanism of epigenetic regulation by histone modification.

## 2 Prostate cancer

**Figure 1:**
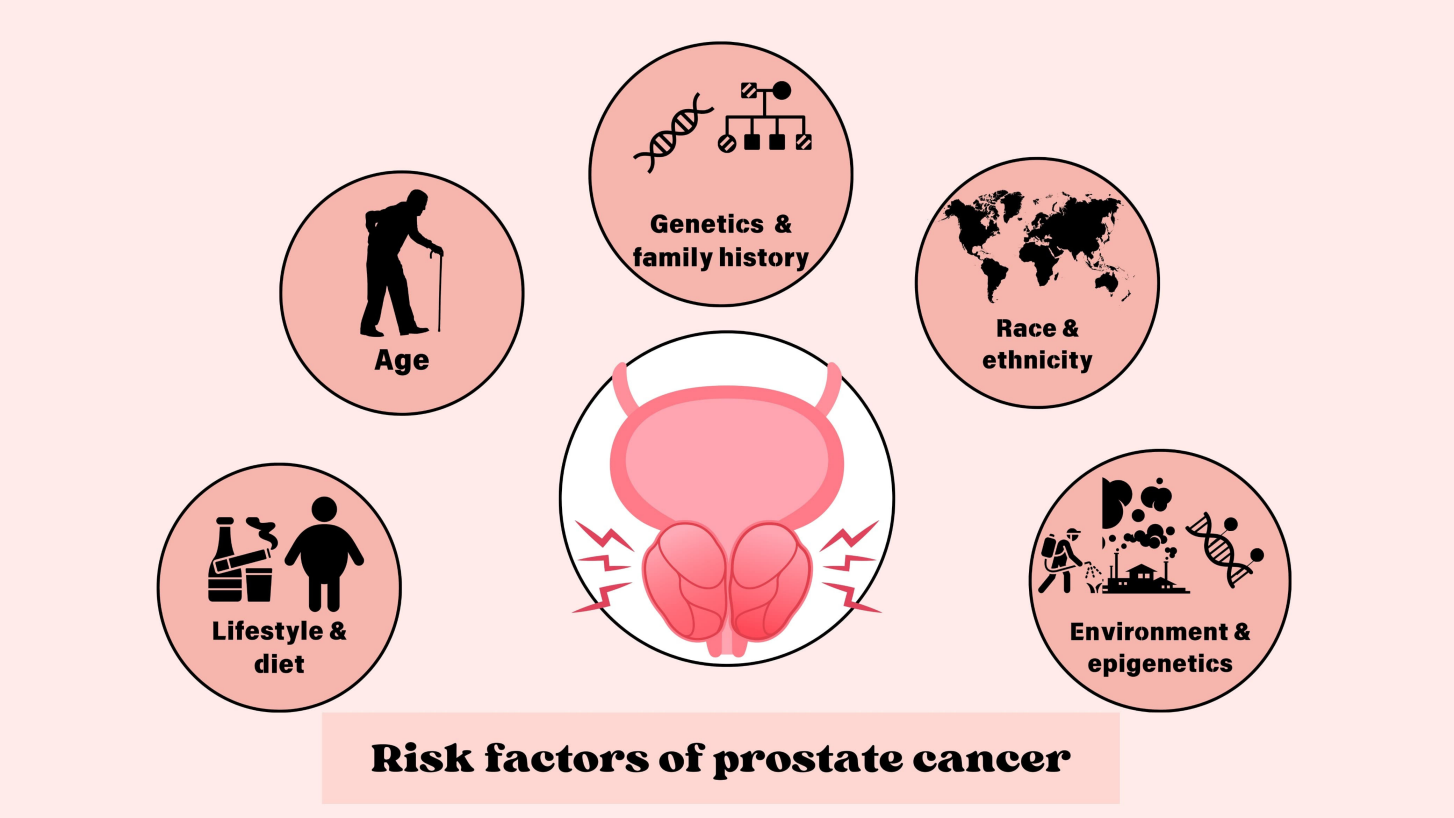
Risk factors of prostate cancer. Major risk factors involved with prostate cancer include age, race and ethnicity, genetics and family history, lifestyle and dietary factors, and epigenetic changes.

Prostate cancer is a type of malignancy that arises in the glandular tissues of the prostate, a small, walnut-shaped organ that contributes significantly to male reproductive functions by producing enzymes, lipids, amines, and metal ions critical for the normal functioning of spermatozoa (11). Prostatic enlargement can be due to benign prostatic hyperplasia (BPH) or prostate cancer, two distinct conditions with different pathological and clinical implications. BPH is a non-cancerous enlargement of the prostate, whereas prostate cancer involves uncontrolled malignant cell growth. BPH typically affects the transitional zone, while prostate cancer often arises in the peripheral zone of the prostate. The progression of prostate cancer involves several stages, beginning with prostatic intraepithelial neoplasia, advancing to localized prostate adenocarcinoma, which may invade nearby tissues, and then ultimately leading to metastatic prostate cancer (12). The predominant type of prostate cancer is adenocarcinoma, although there are other variations also. At the early stage, it remains asymptotic, but as it progresses, symptoms like difficulty in starting urination, interrupted or weak urine flow, frequent urination, mainly during the night, inability to complete emptying of the urinary bladder, burning sensation and cramps during urination, blood in semen or urine, and painful ejaculation may occur. Risk elements associated with it may include age, race and ethnicity, genetics and family history, physical activity and sleep, and other dietary and environmental factors (2) (**Figure 1**).

## 3 Molecular pathophysiology of prostate cancer

The multifaceted nature of prostate cancer arises from the complex interplay of various molecular pathways that govern its initiation and progression. Its complex pathophysiology involves various signaling pathways, genetic alterations, and epigenetic changes such as histone modifications and DNA methylation.

### 3.1 Androgen receptor signaling pathway

**Figure 2:**
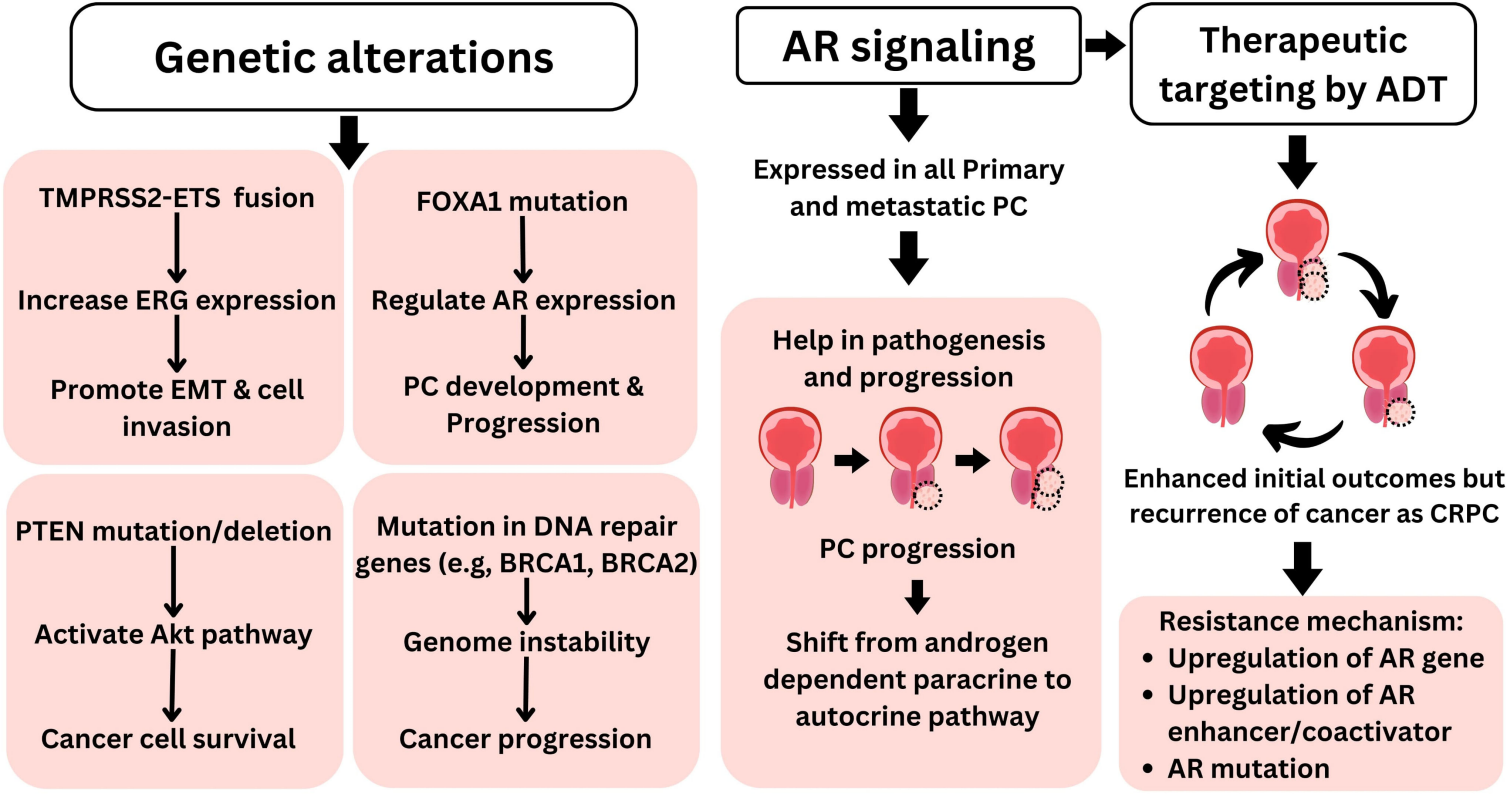
Molecular pathophysiology of prostate cancer. Genetic alterations such as TMPRSS2–ETS fusion, FOXA1 mutation, PTEN deletion/mutation, and defect in DNA repair genes play key roles in prostate cancer pathophysiology. Furthermore, AR signaling plays a central role in prostate cancer pathophysiology, making it suitable therapeutic target for PC. Furthermore, targeting AR with androgen deprivation therapy is among primary therapeutic approaches for PC; however, despite initial favorable outcomes such as reduced tumor size, PC often reoccurs as more lethal CRPC. AR, androgen receptor; PC, prostate cancer; CRPC, castration-resistant prostate cancer.

The androgen receptor (AR), a transcription factor activated by androgens, belongs to the nuclear receptor family. AR plays a significant role in the progression and development of prostate cancer (13). AR is expressed in almost all primary as well as metastatic prostate cancers, irrespective of their grade and stage. This expression is maintained in most androgen-independent and castration-resistant prostate cancer (CRPC). AR signaling continues to be active, promoting the survival and growth of prostate cancer cells (14). The interaction between androgen receptor signaling and the tumor microenvironment is intricate, exhibiting both tumor-promoting and tumor-suppressing effects (15). The role of AR signaling and androgens in metastatic prostate cancer is well understood, demonstrating that prostate cancer cells are highly adaptive at sustaining functional AR signaling to promote cancer growth (16). In prostate cancer, AR signaling in stromal cells, initially high during prostate development, progressively decreases as cancer advances, correlating with worse clinical outcomes and a shift from androgen-dependent paracrine to autocrine pathways in cancer cells. This reduction in stromal AR expression is linked to disease progression, metastasis, and CRPC progression (14). This direct link of AR signaling to prostate cancer makes it a suitable therapeutic target, and androgen deprivation therapy remains the primary treatment option for advanced prostate cancer. These therapies alleviate symptoms, decrease tumor size, and extend patient survival. However, they seldom achieve a cure for the cancer. Prostate cancer frequently returns, leading to lethal CRPC (**Figure 2**). Mechanisms enabling this resistance include upregulation of the androgen receptor gene or enhancer, androgen receptor mutation, variants of the androgen receptor, overexpression of coactivators, and intra-tumoral synthesis of androgens (17). While different mutations in the androgen receptor have been documented in prostate cancer, particular mutations (such as L702H, W742L/C, H875Y, F877L, and T878A/S) are commonly detected following the development of treatment resistance (18). While CRPC progression is generally associated with a gain in AR signaling, a different type of CRPC relies on the loss of AR dependency leading to AR-indifferent conditions such as neuroendocrine prostate cancer (NEPC). NEPC differentiation is primarily associated with a lack of expression of RB1, TP53, and PTEN and upregulation of MYCN and AURKA expression. This transition of PC to NEPC is associated with a key cellular feature called lineage plasticity. It refers to the ability of cancer cells to adapt and change their cellular identity in response to therapeutic pressures such as AR signaling inhibitors (19).

### 3.2 Genetic alterations

Apart from AR mutations, other genetic alterations are also observed in prostate cancer. The most common of these are TMPRSS2–ETS gene fusion, FOXA1 mutation, loss of the PTEN gene, and defects in DNA repair genes (**Figure 2**) (20, 21). In over 50% of prostate tumor cases, TMPRSS2–ETS gene fusion has been documented (20). This fusion involves the androgen-sensitive TMPRSS2 promoter joining with the ERG coding region. In the presence of androgens, this fusion drives increased expression of ERG, significantly influencing cell invasion and the process of epithelial–mesenchymal transition (EMT) (22). FOXA1 is another important transcription factor that is often mutated in multiple types of malignancies including prostate cancer (23). Mutations in FOXA1 are observed in nearly half of primary prostate cancer tumors in Asian men and 20% of tumors in men of other ethnicities (21). FOXA1 is crucial for regulating the expression of numerous genes, particularly AR, during prostate cancer development and progression (24). Moreover, FOXA1 has been identified as an important regulator of alternative splicing in prostate cancer (25). Deletion or mutation of the PTEN gene is found in approximately 20% of the primary prostate cancer samples during radical prostatectomy and up to 50% of CRPC cases (26). PTEN loss leads to the activation of the Akt pathway, enhancing cancer cell survival (20). Furthermore, mutations in genes that code for DNA damage response components, such as BRCA1 and BRCA2, are prevalent in prostate cancer. These mutations diminish the capacity to repair both single- and double-strand DNA damage, thereby compromising the integrity of the genome (27).

### 3.3 Other contributing factors

In addition to AR signaling and genetic alterations, other factors such as inflammation and epigenetics play important roles in the pathophysiology of prostate cancer. Chronic inflammation creates a pro-tumorigenic environment by producing inflammatory cytokines and reactive oxygen species that lead to DNA damage (28). Epigenetic changes, including methylation at DNA and histone modifications, also contribute to the progression of cancer by altering gene expression. The role of epigenetic modifications, in particular histone modifications in prostate cancer, will be discussed in detail in the upcoming section.

## 4 Epigenetic dynamics of prostate cancer

Epigenetic modifications are alterations in the expression of genes that occur independently of any changes to the DNA sequence itself. These epigenetic modifications are heritable and reversible. These modifications commonly include methylation of DNA and the modifications of histone, and these are important for the maintenance of gene expression (29). Epigenetic changes can arise from external environmental factors or exposure to elements such as diet, drugs, and food (30). Epigenetic modifiers are one of the key players in cancer development and progression, making them attractive candidates as prognostic markers and therapeutic targets. These non-mutational epigenetic regulations of gene expression are emerging hallmarks of cancer and are subject to extensive study nowadays (4).

**Figure 3:**
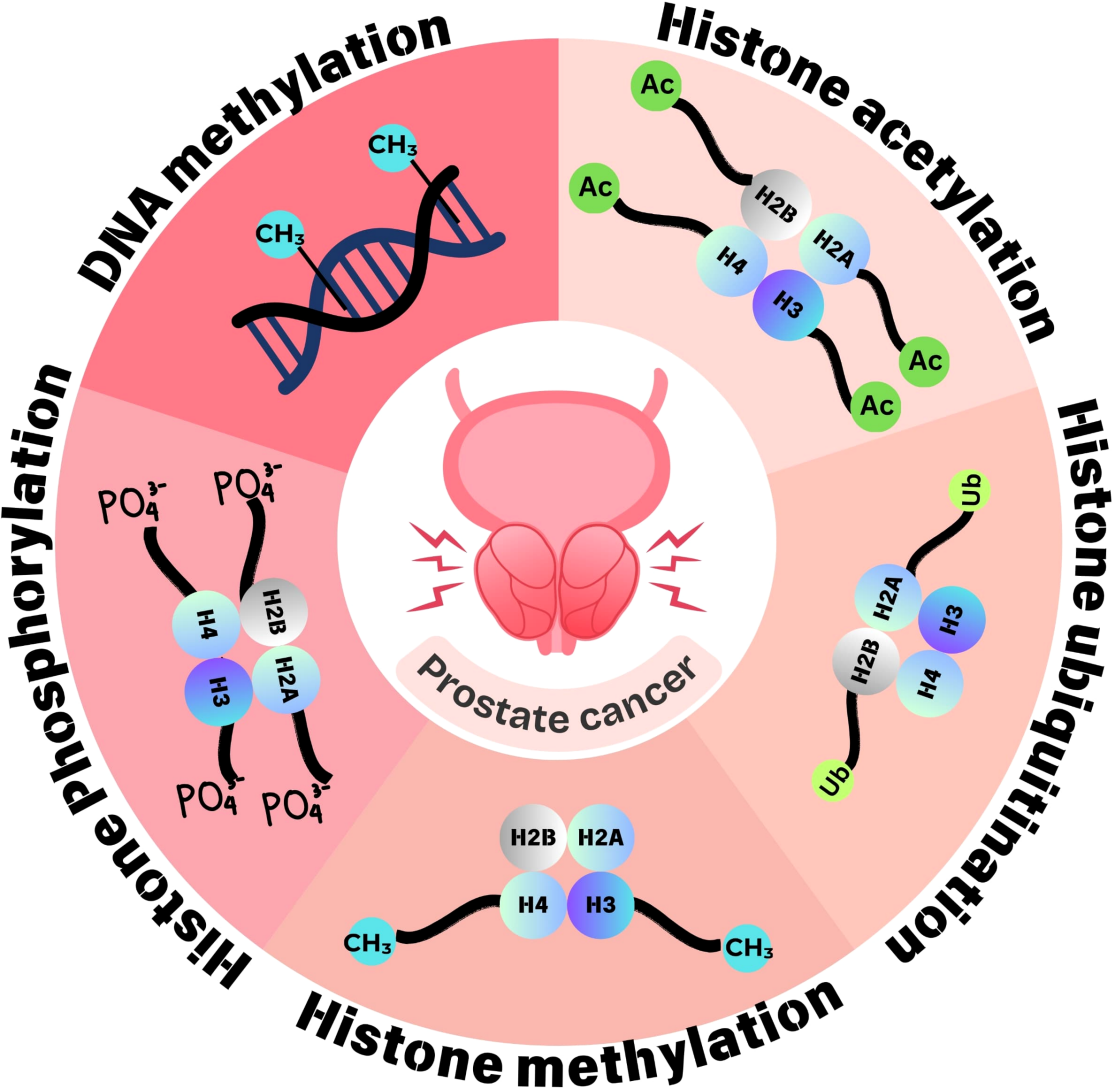
Epigenetic dynamics of prostate cancer. Epigenetic changes implicated in prostate cancer involve aberrant methylation at DNA and histone modifications such as acetylation, methylation, phosphorylation, and ubiquitination. These epigenetic changes regulate gene expression and are thus involved in tumorigenesis and tumor progression.

Epigenetic abnormalities, such as DNA methylation, histone modifications, and remodeling of nucleosomes, happen at all stages of the development and advancement of prostate cancer (31) (**Figure 3**). These epigenetic markers can be used as biomarkers for both the diagnosis and prognosis of prostate cancer (32). The methylation of DNA is a fundamental epigenetic mechanism that regulates gene expression and various cell processes. It refers to the addition of methyl group to DNA molecules, specifically at cytosine bases, typically occurring at CpG dinucleotides, and this modification is catalyzed by enzyme group DNA methyltransferases (33). Abnormal DNA methylation patterns, especially CpG island hypermethylator phenotype, are associated with specific clinical characteristics and outcomes in prostate cancer. These include tumor aggressiveness, high Gleason scores, elevated PSA level, advanced stages, poorer prognosis, and reduced survival rates. Commonly hypermethylated genes in prostate cancer are involved in functions such as apoptosis, DNA damage repair, cell cycle regulation, cell adhesion, hormonal responses, and signal transduction. Examples include GSTP1, RARβ, RASSF1A, CDH13, APC, DAPK, p16, FHIT, CDH1, and MGMT (5). Another epigenetic phenomenon related to prostate cancer is DNA hypomethylation, which involves the removal of methyl groups from CpG sites that are typically methylated, leading to increased expression of genes. In prostate cancer, hypomethylation influences genes encoding urokinase-type PLAU (PLAU and its receptor are crucial for tumor invasion and metastasis development by breaking down the extracellular matrix), HPSE (a versatile protein involved in extracellular matrix degradation and heparan sulfate chain breakdown of proteoglycans), and CYP1B1 (essential for estrogen metabolism and activation of procarcinogens) (34).

In addition to DNA methylation, histone modifications like histone acetylation and histone methylation are pivotal in regulating gene expression and influencing cancer development and progression. These histone modifications are briefly discussed in the proceeding sections.

## 5 Histone modifications in prostate cancer

**Figure 4:**
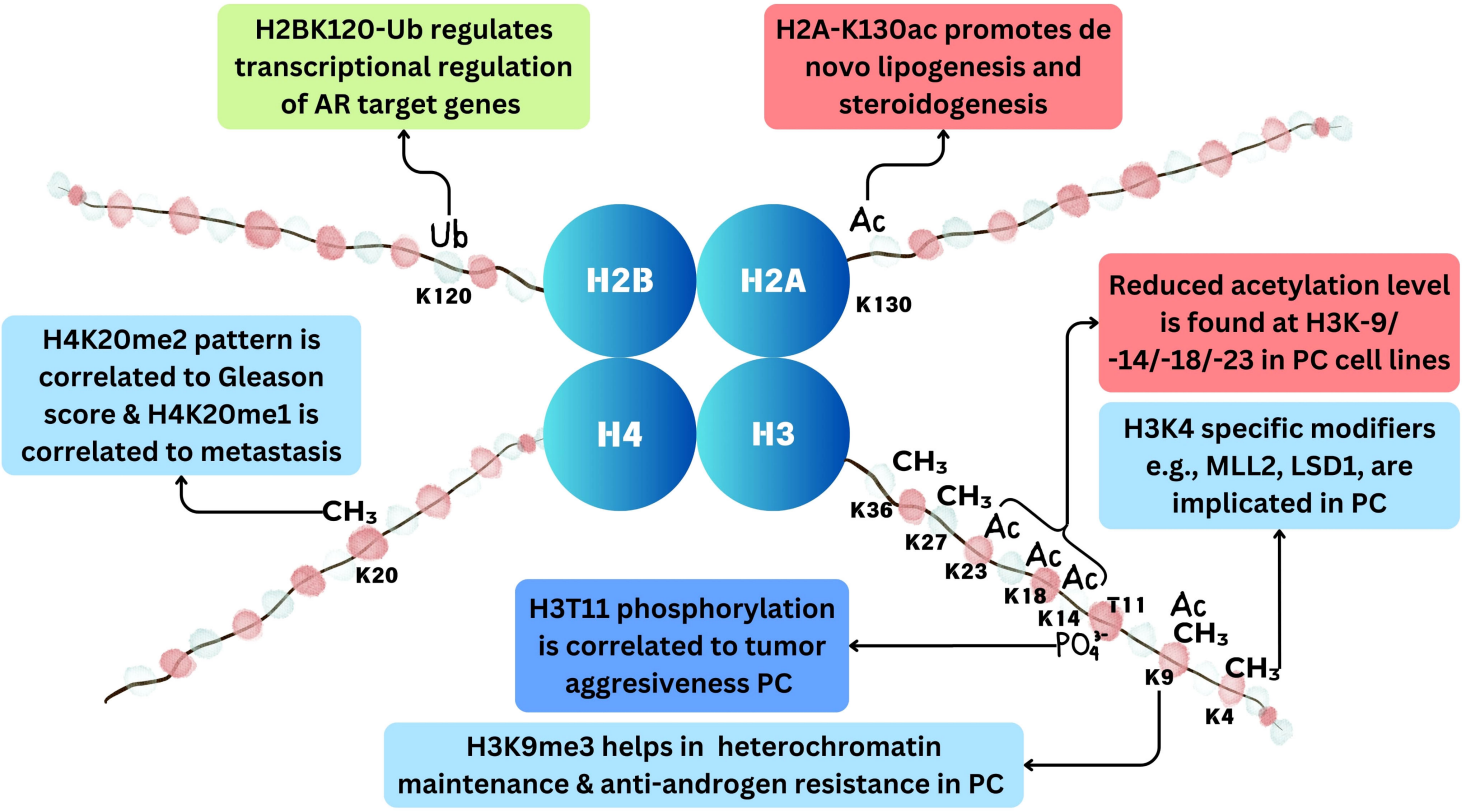
Histone modifications in prostate cancer. Methylation at histone occurs at H3K4, H3K9, H3K27, H3K36, and H4K20 positions. Acetylation occurs at H3K9, H3K14, H3K18, H3K23, and H2AK130 positions. Ubiquitination occurs at H2BK120 position and phosphorylation at H3T11 position in prostate cancer.

Among epigenetic changes, histone modifications (histone acetylation, histone methylation, etc.) play a crucial role in gene expression regulation, and their dysregulation has been implicated in cancer development and progression (35). Post-translational modifications, including methylation, phosphorylation, acetylation, sumoylation, deamination, ubiquitylation, proline isomerization, ADP-ribosylation, and β-*N*-acetylglucosamine, can affect the projecting tail of histones. Histone modification influences DNA replication, repair, and recombination processes as well as chromatin structure regulation and remodeling (36). Histone modifications and differential expression of histone modifiers are reported in different cancers including prostate cancer (**Figure 4**). The process of addition of an acetyl group to lysine amino acid residue in the projecting tail of histone is known as histone acetylation. Usually linked to transcriptional activity, it is regulated by two distinct sets of enzymes: HDACs, which eliminate acetyl groups, and HATs, which add them (36). Histone methylation is another post-translational modification process that refers to the methyl group addition to the histone proteins. Histone methylation occurs on all basic residues: arginine, lysine, and histidine. Among these, lysine may undergo mono-methylation, di-methylation, or tri-methylation, whereas arginine may undergo mono-methylation, symmetrical di-methylation, or asymmetrical di-methylation, and histidine could be mono-methylated, although this is relatively rare (37). *S*-Adenosyl methionine, the methyl-donating substrate of histone methyltransferases, mediates histone methylation (38). Apart from acetylation and methylation, histone ubiquitination (removal or addition of ubiquitin at the C-terminal end of histone H2A and H2B) and ubiquitin-activating enzymes are also associated with prostate cancer (34). Other histone modifications implicated in cancer include phosphorylation and ADP-ribosylation (35).

### 5.1 Histone acetylation in prostate cancer

As discussed earlier, histone acetylation refers to the acetyl group addition to lysine amino acid residues at histone tails, and it is regulated by two opposing enzyme groups: HATs and HDACs. HATs (often referred to as writers), using acetyl CoA as a cofactor, add acetyl moiety at the ϵ-amino group of lysine residue; this modification weakens the interaction between histone and DNA, leading to transcriptional activation. However, HDACs, also known as erasers, are enzymes that remove the acetyl moiety from the lysine amino acid residues; this interaction leads to the condensation of chromatin and finally transcriptional repression (36). Bromodomains, also known as “readers”, are tiny protein modules that read the acetylation on lysine amino acid residues (39).

The acetylation level of histone H3 at the N-terminal end at different positions such as lysine 9, lysine 14, lysine 18, and lysine 23 is reduced in cancer cell lines such as PC-3, LNCaP, DU-145, and clinical prostatic adenocarcinomas, in comparison to that in the non-cancerous prostate cell lines such as RC170N/h and RC165N/h. This reduced acetylation is associated with significantly elevated HDAC activity in cancer cells. Inhibiting HDACs restores the acetylation of histone and increases the expression of the p21 gene, highlighting that increased HDAC activity underlies the deficient histone acetylation seen in prostate cancer (40). Furthermore, a recent study by Nguyen et al. revealed a novel mechanism including histone acetylation by which prostate cancer cells adapt to androgen deficiency through dual phosphorylation of sterol regulatory element-binding protein 1 (SREBF1) at Y673 and Y951. Dual-phosphorylated SREBF1 senses low androgen levels and dissociates from AR, enabling its nuclear translocation. In the nucleus, SREBF1 recruits KAT2A/GCN5, leading to the acetylation of H2A at lysine 130. This epigenetic marker promotes the transcription of genes necessary for *de novo* lipogenesis and steroidogenesis (41).

Furthermore, dysregulation of HAT and HDAC expression is often observed in prostate cancer. Histone acetyltransferases such as p300 and CBP are prominent coactivators of androgen receptors and have a pro-tumor role in prostate cancer (34). A recent study reported that histone acetyltransferase 1 (HAT1) is upregulated in prostate cancer cells and correlated to disease progression to CRPC. The same study further reported that HAT1 knockdown re-sensitizes the drug response to CRPC cells (42). Similarly, histone deacetylase is also found to be implicated in prostate cancer, and the use of histone deacetylase inhibitors has shown enhanced therapeutic outcomes (43, 44).

Different histone acetyltransferases and histone deacetylases implicated in prostate cancer are summarized in **Table 1**.

**Table 1:** Different HATs and HDACs implicated in prostate cancer.

Class	Name	Expression pattern	Methodology	Clinical association	Survival impact (p-value)	Patient cohort data	Reference
HATs	KAT2A	Upregulated in high-grade disease as well as in abiraterone-resistant prostate cancer C4-2 cells	GEO, TCGA, Oncomine, IHC, Western blotting	Higher expression correlates with worse prognosis, biochemical relapse, and high Gleason score (≥8)	OS (p < 0.05), DFS (p < 0.0001), PFS (p < 0.001)	12 PC patients for IHC validation	(45)
	KAT2B	Upregulated in high-grade PC tissue as compared to low-grade and BPH tissue; regulates NLRP3 acetylation	GEO, TCGA, Oncomine	Involved in AR regulation of inflammation and cell growth	Not specified	C4-2 prostate cancer cells (three independent experiments)	(46)
	HAT1	Expression in PC tissues was higher than that in non-tumor prostate tissues	TCGA, IHC, RNA-seq, functional studies	HAT1 promotes PC and castration-resistant prostate cancer (CRPC) progression by upregulating AR (including AR-V7) via a BRD4-mediated pathway; knockdown re-sensitizes CRPC cells to ENZ	Not specified	LNCaP, C4‐2, and 22Rv1 human prostate cancer cell lines	(42)
	KAT5	Significantly reduced in prostate cancer tissues and cell lines	Oncomine, Human Protein Atlas, RT-PCR, Western blotting, Kaplan–Meier Plotter	Low KAT5 expression correlates with tumor progression; overexpression reduces PC cell proliferation and induces apoptosis via Bax, cytochrome *c*, caspase 3 activation, and PARP cleavage	OS (p < 0.01), DFS (p < 0.001)	LNCaP cell model, prostate adenocarcinoma samples (IHC validation)	(47)
	TAF1	Increased levels of TAF1 expression with prolonged hormone treatment and CRPC progression	Tissue microarray, Co-IP, Western blotting, IHC, GST pulldown assay, ChIP assay, transcriptional assays, ubiquitination assay	Interacts with AR via HAT and E1/E2 domains to enhance AR transcriptional activity and ubiquitinates AR	Not specified	112 prostate cancer samples stratified by androgen withdrawal therapy: no treatment (n = 21), <3 months (n = 21), 3–6 months (n = 28), >6 months (n = 28), CR tumors (n = 14)	(48)
HDACs	HDAC1	HDAC1 overexpression is associated with adverse tumor features		–	–	–	(49)
	HDAC 1, 2, and 3	Highly expressed in PC, class I HDAC expression in prostate tissue; HDAC1 and HDAC2 positively correlate with Gleason score (HDAC1, p = 0.006; HDAC2, p = 0.047; HDAC3, p = 0.584) and Ki-67 proliferative index	IHC, survival analysis, Kattan nomogram	High HDAC1 and HDAC2 expression correlate with higher-grade tumors and lower DFS probability; HDAC2 shows significant correlation with relapse-free survival, particularly in Gleason 7 tumors	DFS: HDAC1 (p = 0.203), HDAC2 (p = 0.036), HDAC3 (p = 0.946)	192 prostate cancer patients (age 46–73 years, median 62.5 years)	(50)
	HDAC4 and 5	HDAC4: overexpressed in primary and recurrent prostate cancer compared to benign tissues. Higher expression in androgen-independent cell lines (PC3, DU145, and LNCaP-AI) compared to androgen-dependent cells HDAC5: overexpressed in recurrent prostate cancer compared to primary PC (p = 0.004). Highest expression in LAPC4 cells (androgen-dependent)	Microarray analysis, *in situ* hybridization, immunohistochemistry, real-time RT-PCR, Western blotting analysis, cell culture, cell proliferation, and Matrigel invasion assays	Increased HDAC4 expression in androgen-independent cell lines suggests a role in prostate cancer progression and therapeutic resistance Higher HDAC5 expression in recurrent prostate cancer suggests a role in disease progression	Not specified	98 prostate cancer samples; PC3, DU145, and LNCaP-AI cell lines	(51)
	HDAC11	Expressed in prostate cancer; downregulation in CAR-T cells enhances anti-tumor activity	Human Protein Atlas, FACS analysis, CFSE assay, functional studies	Enhanced CAR-T cell efficacy against prostate cancer via increased activation, cytotoxicity, and memory cell formation	Not specified	Prostate cancer cell lines PC-3 (CRL-1435), DU-145 (HTB-81)	(52)
	SIRT1	Upregulated in prostate cancer cell lines and tissues	Western blotting, RT-PCR, IHC, functional studies	Functions as an oncogene by inhibiting FOXO1 acetylation and transcription and promoting prostate cancer progression	Not specified	Prostate carcinoma cell lines (viz. LNCaP, 22Rν1, DU145, and PC3)	(53)
	SIRT2	Increased expression in CRPC and neuroendocrine prostate cancer (NEPC)	TCGA, GSE54460, qPCR, IHC, functional studies	Promotes cell proliferation and S-phase progression and reduces apoptosis. Activates ERK1/2 pathway and induces lactosylceramide production via B4GALT5 upregulation, enhancing migration and invasion	Not specified	TCGA cohort and GSE54460 cohort	(54)
	SIRT2	Decrease in both primary prostate cancer and metastatic PC samples	TCGA, GEO, IHC, tissue microarrays	Decreased SIRT2 correlates with higher-grade cancer and shorter PSA recurrence time	PSA recurrence time: high H3K18Ac (350 days) vs. low H3K18Ac (1542 days) (p = 0.03)	71 radical prostatectomy patients (IHC, tissue microarrays), TCGA (n = 499), GEO (n = 504)	(55)
	SIRT3	Moderately downregulated in human prostate carcinoma	Oncomine Database (multiple microarray datasets), IHC staining	Positively correlates with patient survival	High SIRT3 copy number is associated with significantly longer overall survival (Kaplan–Meier analysis)	19 human clinical specimens (Oncomine), 96 human samples (Oncomine), 109 patient samples (IHC: 32 benign, 77 tumor tissues)	(56)
	SIRT4	Downregulated in human prostate cancer tissues	Immunohistochemistry, Western blotting, qRT-PCR, GEPIA database analysis	Lower SIRT4 expression is significantly associated with higher Gleason scores, indicating more aggressive disease	Not specified	89 prostate cancer patients; 492 prostate cancer samples and 52 normal prostate tissues from the TCGA database	(57)
	SIRT6	Higher mRNA levels of SIRT6 were observed in the PC samples compared with those of the normal tissue	Generation of lentiviruses, Western blotting, RT-PCR analysis, cytotoxic assay, flow cytometry, Kaplan–Meier analysis, log-rank test	Higher expression of SIRT6 is significantly associated with higher Gleason scores	Associated with poor OS (p = 0.017)	TCGA cohort	(58)

HATs, histone acetyltransferases; HDACs, histone deacetylases; IHC, immunohistochemistry; PC, prostate cancer; BPH, benign prostatic hyperplasia; CRPC, castration-resistant prostate cancer; PSA, prostate-specific antigen; ChIP, chromatin immunoprecipitation; GEO, Gene Expression Omnibus; TCGA, The Cancer Genome Atlas; OS, Overall Survival; DFS, Disease-Free Survival; PFS, Progression-Free Survival; ENZ, Enzalutamide; Co-IP, Co-Immunoprecipitation; FACS, Fluorescence-Activated Cell Sorting; CFSE, Carboxyfluorescein Succinimidyl Ester; GEPIA, Gene Expression Profiling Interactive Analysis.

### 5.2 Histone methylation in prostate cancer

Histone methylation is another epigenetic marker associated with prostate cancer, and distinct patterns of gene expression of HMTs and HDMs have been reported in different studies. Histone methylation refers to the addition of methyl group to basic amino acid residue (arginine, lysine, and histidine) of the histone chain. It is mediated by *S*-adenosyl methionine, which acts as a methyl-donating substrate (59). Some of the most studied histone methylation patterns include H3K4 methylation (i.e., methylation at lysine 4 residue of histone H3), H3K9 methylation (i.e., methylation at lysine 9 of histone H3), H3K27 methylation (histone H3 lysine 27 methylation), H3K36 methylation (histone H3 lysine 36 methylation), and H4K20 methylation (histone H4 lysine 20 methylation) (60, 61). These methylation patterns can be associated with either transcription activation (H3K4me3) or repression (H3K9me2/3), thus modulating key cellular processes (61). Methylation, in contrast to acetylation, does not change the histone protein’s net charge. Instead, methylation recruits different effector proteins, referred to as histone methylation readers, to cause the transcriptional alteration (60).

A number of studies have reported differential methylation patterns and dysregulated expression of HDMs and HMTs in prostate cancer. Pang et al. reported that androgen-stimulated H3K4me2 methylation in prostate cancer is mediated by PI3 K/p110beta-dependent signaling, which may be exploited as a new biomarker for disease prognosis and targeted therapy (62). MLL2 is an important H3K4 methyltransferase that is involved in the activation of the PI3K/EMT process and also induces DNA damage in prostate cancer (61). SMYD3 is another HMT that is found to be implicated in prostate tumors. Its downregulation in prostate cancer inhibits cancer development by suppressing the transcription activation of cyclin D2 or AR (63). Among H3K4 demethylases, LSD1 directly suppresses androgen receptor transcriptional activity through H3K4 demethylation (64). Additionally, LSD1 can enhance CRPC by controlling mitotic kinesin and the centromere-binding protein CENPE (65). Another H3K4 HDM, KDM5D, is also implicated in androgen receptor signaling and thus prostate cancer (66). Furthermore, H3K9me3 is critical for the maintenance of heterochromatin and the development of anti-androgen resistance in prostate cancer, and the methylation of H3K9 by EHMT1 has been linked to poor patient outcomes from hormone therapy. Also, H3K9me3 writers’ inhibition suppresses anti-androgen resistance (67). A 2017 study highlighted that H3K27me3 is a crucial epigenetic modification in the progression of prostate cancer (68). Furthermore, H3K27- and H3K36-specific HMTs and HDMs are also implicated in prostate cancer (69). Among H4 methylation sites, methylation at H4K20me2 has shown a significant difference in tumor vs. normal tissues. H4K20me1 pattern is found to significantly differentiate CRPC tissues from other kinds of prostate tissues. Additionally, a significant correlation between H4K20me1 with lymph node metastases and H4K20me2 Gleason score was found (70). Different HMTs and HDMs implicated in prostate cancer are summarized in **Table 2**.

**Table 2:** Different HMTs and HDMs implicated in prostate cancer.

Class	Name	Expression pattern	Methodology	Clinical association	Survival impact (p-value)	Patient cohort data	References
HMTs	SETD1A	Upregulated in prostate tumors than that in normal prostate tissue	Gene expression analysis, qRT-PCR, chromatin immunoprecipitation (ChIP), and functional assays in C4-2B and LNCaP cells	Promotes proliferation, migration, invasion, and cancer stem cell formation by activating FOXM1	High SETD1A expression correlates with low survival rates in prostate cancer patients (GSE40272)	LNCaP, PC-3, DU145, and LNCaP-LN3 cell lines	(71)
	EZH	Upregulated in PC tissue as compared to normal	RT-qPCR	Higher expression in high Gleason score (GS) (p = 0.048)	Significant association with DFS	160 patients with clinically localized prostate adenocarcinoma	(72)
	KMT2A	Downregulated in PC tissue as compared to normal	RT-qPCR	Higher expression in pT3b cases (p = 0.041)	Not specified		(72)
	KMT2B	Downregulated in PC tissue as compared to normal	RT-qPCR	No significant association with clinical stage or GS	Not specified		(72)
	KMT2C	Downregulated in PC tissue as compared to normal	RT-qPCR	Increased expression in high Gleason score (p = 0.018)	Not specified		(72)
	KMT2D	Downregulated in PC tissue as compared to normal	RT-qPCR	No significant association with clinical stage or GS	Not specified		(72)
	SMYD3	Upregulated in PC tissue as compared to normal	RT-qPCR	Higher expression in pT3b cases (p = 0.044), retained prognostic significance in multivariate analysis	Significant association with DFS		(72)
	SUV39H2	Upregulated in PC tissue as compared to normal	RT-qPCR	No significant association with clinical stage or GS	Not specified		(72)
	PRMT6	Upregulated in PC tissue as compared to normal	RT-qPCR	Showed highest diagnostic potential (AUC = 0.923, sensitivity 90.0%, specificity 73.3%)	Not specified		(72)
	SMYD2	Significantly upregulated in PC tissues and cell lines	qRT-PCR, Western blotting, IHC, functional assays (proliferation, migration, invasion, and EMT), SMYD2 knockdown, overexpression studies, mouse models	High SMYD2 expression linked to poor CRPC-free survival and overall survival; promotes drug resistance in CRPC	High SMYD2 expression associated with poor prognosis	–	(73)
HDMs	KDM1A	Significantly high expression in tumor tissue as compared to benign prostate tissue	–	–	–	–	(74)
	KDM5A	Upregulated in PC tissue as compared to normal	RT-qPCR	No significant association with clinical stage or GS	Not specified	160 patients with clinically localized prostate adenocarcinoma	(72)
	KDM6A	Upregulated in PC tissue as compared to normal	RT-qPCR	No significant association with clinical stage or GS	Not specified		(72)
	KDM7A	Significantly upregulated in prostate cancer tissue	–	–	–	–	(75)
	KDM4C	A much higher expression in prostate carcinoma as compared to paired adjacent normal prostate tissues					(76)
	KDM5C	Upregulated in prostate cancer	IHC on TMA Western blotting validation	Higher nuclear KDM5C correlates with lower Gleason scores (p = 0.004) Cytoplasmic expression not significantly associated with clinical parameters	High nuclear KDM5C correlates with better biochemical recurrence-free survival (p = 0.027)	Bonn cohort: 262 patients Berlin cohort: 560 patients PSA relapse: Bonn 19.1% (44/230), Berlin 17.1% (91/531)	(77)

HMTs, histone methyltransferases; HDMs, histone demethylases; PC, prostate cancer; EMT, epithelial–mesenchymal transition; CRPC, castration-resistant prostate cancer; PSA, prostate-specific antigen; AUC, Area Under the Curve; TMA, Tissue Microarray.

### 5.3 Other histone modifications in prostate cancer

Other important histone modifications implicated in cancer include phosphorylation and ubiquitination. Histone phosphorylation happens at threonine and serine amino acid residues. This process is modulated by kinase and phosphatase. Phosphorylation, like acetylation, leads to the opening of the chromatin structure by negative charge addition to the histone and also leads to the recruitment of effector proteins (69). In prostate cancer, PRK1 phosphorylates histone H3 at threonine 11 (H3T11) upon AR activation, facilitating AR-dependent transcription by promoting histone demethylation and RNA polymerase II recruitment. Elevated PRK1 levels and phosphorylated H3T11 correlate with prostate cancer aggressiveness, suggesting PRK1 inhibition as a potential therapeutic strategy for blocking AR-induced tumor proliferation (78).

Histone ubiquitination is another post-translational modification where ubiquitin molecules are covalently attached to histone proteins within chromatin (79). This modification primarily occurs on histone H2A and histone H2B. Histone ubiquitination regulates various chromatin-related processes, including transcriptional regulation, DNA repair, and chromatin compaction (80). Histone ubiquitination, specifically at H2BK120 regulated by RNF20 and RNF40, plays a crucial role in the transcriptional regulation of AR target genes in prostate cancer. The dynamic nature of H2BK120-ub and its interplay with other histone modifications are important for gene expression regulation. Variations in RNF20 expression and its interaction with AR play a complex role in prostate cancer progression, offering potential targets for therapeutic intervention (10).

## 6 Therapeutic implications of histone modifications in prostate cancer

As we have discussed in previous sections, epigenetic modifications play an important role in prostate cancer pathogenesis and progression. In this section, we will discuss the therapeutic implications of targeting epigenetic modifications in prostate cancer, exploring various strategies and their potential benefits. Epigenetic modifiers such as HATs, HDACs, HMTs, and HDMs play key roles in prostate cancer progression through various mechanisms, and targeting these modulators has shown significant therapeutic outcomes (81).

HAT inhibitors are being explored as potential therapeutic agents in prostate cancer, particularly in the context of more lethal CRPC. Presently, several inhibitors targeting HATs have been explored and shown therapeutic effects against different cancers such as breast cancer, Renal Cell Carcinoma (RCC), bladder cancer, and prostate cancer (82). CCS1477, a novel small-molecule inhibitor targeting the bromodomain of histone acetyltransferases p300 and CBP, demonstrates antitumor activity by inhibiting AR and C-MYC signaling in CRPC. It shows potential in reducing AR signaling and affecting metastatic CRPC target expression in clinical settings (83). Furthermore, various HDAC inhibitors are also in clinical trials for different cancers including prostate cancer. Several HDAC inhibitors, such as vorinostat (SAHA), belinostat (PXD-101), panobinostat (LBH589), chidamide (CS055, HBI-8000), and romidepsin (FK228), have been approved by the U.S. Food and Drug Administration (FDA) as medicines for the treatment of skin T-cell lymphoma (TCL) and peripheral TCL (79). Hematological malignancies have demonstrated encouraging responses to HDAC inhibitors. However, HDAC inhibitors have not successfully passed clinical trials for solid tumors, even though there have been promising results in biological studies, preclinical research, and early clinical trials. For instance, clinical trials involving vorinostat (SAHA) have shown drug toxicity and no promising results in clinical outcomes (84).

Furthermore, inhibitors of HMTs and HDMs are also explored for their possible therapeutic benefits in different cancers including prostate cancer. One of the most thoroughly researched histone methylation inhibitors is the class of EZH2 inhibitors. Blocking EZH2 has shown promising outcomes, especially in lymphomas; however, targeting EZH2 has not yet demonstrated significant clinical activity in solid tumors (85). Among SMYD family HMTs, SMYD3 inhibition by inhibitor molecule BCI-121 has shown decreased proliferation in colon cancer cell lines (86). Among inhibitors of HDM, LSD1 inhibitors are the most extensively studied, and many LSD2 inhibitors such as tranylcypromine (TCP), ORY-1001 iadademstat, and bomedemstat are already in phase 1/2 of clinical trials, but mostly in breast cancer and leukemia (87). In prostate cancer, pargyline (an inhibitor of LSD1) has also shown promising therapeutic potential. Pargyline decreased the migration and invasion capabilities of LNCap cells and hindered the EMT process by upregulating E-cadherin expression while downregulating N-cadherin and vimentin expressions both *in vitro* and *in vivo*. Additionally, pargyline delayed the transition of prostate cancer from an androgen-dependent state to an androgen-independent one (88).

In conclusion, targeting histone modifications offers promising therapeutic potential for prostate cancer, particularly in overcoming treatment-resistant forms like CRPC. Ongoing research and clinical trials are crucial to optimize these strategies.

## 7 Conclusion and future prospects

Histone modifications are pivotal in the regulation of gene expression in prostate cancer, influencing various aspects of the disease’s progression and resistance to therapy. The dysregulation of histone acetylation and methylation, along with other modifications, has been implicated in critical processes such as DNA damage repair, cell cycle regulation, and hormone response. These modifications provide valuable biomarkers for the diagnosis and prognosis of prostate cancer, highlighting the potential for personalized treatment approaches. Although significant advancements have been made in epigenetic biomarkers and their association with prostate cancer, several challenges remain in their clinical transition. These include evaluating the role of epigenetic alterations in normal biological processes and disease progression and interpreting large-scale epigenetic data to ensure robust validation of biomarkers.

Current research emphasizes the importance of developing therapies that target specific histone modifications and the enzymes that regulate them, such as HDACs, HATs, HMTs, and HDMs. The therapeutic potential of these targets is particularly promising in addressing treatment-resistant forms of prostate cancer, such as CRPC. Future studies should focus on further elucidating the role of these modifications in cancer biology and exploring novel inhibitors that can selectively modulate these epigenetic changes. By advancing our understanding of histone modifications in prostate cancer, we can pave the way for more effective, targeted therapeutic strategies that improve patient outcomes and address the complexities of this disease.
